# Transcriptomic and proteomic data provide new insights into cold-treated potato tubers with T- and D-type cytoplasm

**DOI:** 10.1007/s00425-022-03879-2

**Published:** 2022-04-05

**Authors:** Katarzyna Szajko, Dorota Sołtys-Kalina, Małgorzata Heidorn-Czarna, Paulina Smyda-Dajmund, Iwona Wasilewicz-Flis, Hanna Jańska, Waldemar Marczewski

**Affiliations:** 1 Plant Breeding and Acclimatization Institute-National Research Institute, Platanowa 19, 05-831 Młochów, Poland; 2 Faculty of Biotechnology, University of Wroclaw, 50-383 Wrocław, Poland

**Keywords:** Cold-sweetening, Cytoplasm types, Gene expression, Potato tubers, Protein expression

## Abstract

**Main conclusion:**

Tuber-omics in potato with the T- and D-types of cytoplasm showed different sets of differentially expressed genes and proteins in response to cold storage.

**Abstract:**

For the first time, we report differences in gene and protein expression in potato (*Solanum tuberosum* L.) tubers possessing the T- or D-type cytoplasm. Two F1 diploid reciprocal populations, referred to as T and D, were used. The pooling strategy was applied for detection of differentially expressed genes (DEGs) and differentially expressed proteins (DEPs) in tubers consisting of extreme chip colour after cold storage. RNA and protein bulks were constructed from contrasting phenotypes. We recognized 48 and 15 DEGs for the T and D progenies, respectively. DEPs were identified in the amyloplast and mitochondrial fractions. In the T-type cytoplasm, only 2 amyloplast-associated and 5 mitochondria-associated DEPs were detected. Of 37 mitochondria-associated DEPs in the D-type cytoplasm, there were 36 downregulated DEPs in the dark chip colour bulks. These findings suggest that T- and D-type of cytoplasm might influence sugar accumulation in cold-stored potato tubers in different ways. We showed that the mt/nucDNA ratio was higher in D-possessing tubers after cold storage than in T progeny. For the D-type cytoplasm, the pt/nucDNA ratio was higher for tubers characterized by dark chip colour than for those with light chip colour. Our findings suggest that T- and D-type cytoplasm might influence sugar accumulation in cold-stored potato tubers in different ways.

**Supplementary Information:**

The online version contains supplementary material available at 10.1007/s00425-022-03879-2.

## Introduction

Plant cells possess two membrane enclosed organelles, mitochondria and plastids (chloroplasts). They enable essential roles in cellular homeostasis during plant growth and development and response to stresses. It is estimated that 3000–4000 different proteins are present inside plant chloroplasts (Fey et al. 2005). More than 2000 different proteins were calculated to be in higher plant mitochondria (Rao et al. 2017). Although plant mitochondria and plastids contain their own genomes, the majority of organellar proteins are encoded in the nucleus (Blanco et al. 2014; Mackenzie and Kundariya 2019). There is close communication between organelles and the nucleus via antergrade (nucleus-to-organelles) and retrograde (organelles-to-nucleus) signalling pathways (Crawford et al. 2018; Wu et al. 2019). The retrograde response is connected with signals emitted from the organelles that modulate the expression of nuclear genes (Pfannschmidt et al. 2020; Wang et al. 2020). Plastid–nuclear complexes and stromules (stroma-filled tubular plastid extensions) are postulated to facilitate transfer of signalling molecules, including proteins, from plastids to the nucleus in response to environmental changes (Mullineaux et al. 2020). Changes in the expression of mitochondrial proteins in response to stress conditions initiate a signalling cascade that can modulate expression of nuclear genes (Wang et al. 2020). Decrease in respiratory energy production involves perturbations in plant cell physiology (Welchen et al. 2021). To adapt to stress conditions, degradation of proteins, protein aggregates and whole organelles by an autophagy-dependent mechanism is activated (Avin-Wittenberg 2019).

Potato (*Solanum tuberosum* L.) is the third-most important crop worldwide. Carbohydrate metabolism in stored potatoes is mainly under control of enzymatic and nonenzymatic factors involved in the pathways of starch synthesis and degradation, glycolysis, hexogenesis and mitochondrial respiration. When potato tubers are stored after harvest at temperature above 10 °C, their natural dormancy usually lasts 2–3 months depending on the variety. Storage tubers at lower temperature (4–6 °C) reduce water loss, sprouting and disease. The adaptation of tubers to low temperature involves a wide range of physiological processes including starch–sugar interconversion. Amyloplasts are known to play an essentially important role during starch mobilization. Mitochondria are involved in the processes of organic acid metabolism via the tricarboxylic acid (TCA) cycle and cytochrome-mediated and alternative pathway respiration (Sowokinos 2001; Blenkinsop et al. 2004; Zhang et al. 2017). Based on PCR marker system the T-, D-, P-, A-, M-, and W-cytoplasm types were found in the common potato gene pool (Hosaka and Sanetomo 2012).

Cold accelerates the conversion of tuber starch into reducing sugars, i.e., glucose and fructose. This phenomenon is known as cold-induced sweetening (CIS) (Isherwood 1973) and protects plants from cold stress, but such potato tubers are unsuitable for processing. Specifically, thermal processing generates a nonenzymatic Maillard reaction, which results in dark-pigmented French fries and chips that are not accepted by consumers. The reducing sugar content in potato tubers is a quantitative trait. Quantitative trait locus (QTL) mapping revealed different sets of chromosome regions that significantly affect the reducing sugar content in tubers after harvest and cold storage (Werij et al. 2012; Sołtys-Kalina et al. 2015, 2020). Positive maternal effect on the glucose content in tubers from diploid potato reciprocal crosses was observed (Jakuczun and Zimnoch-Guzowska 2004). Significant differences in total tuber proteins between CIS-tolerant and CIS-sensitive potato cultivars were identified by comparative proteomics and association mapping analyses (Fischer et al. 2013).

Transcript abundance can only partially explain protein abundance. There is a need for the integration of omics approaches to obtain a more complete picture for metabolism and function of living organisms (Zapalska-Sozoniuk et al. 2019). The D-type cytoplasm is mostly prevalent in potatoes derived from *S. demissum*, while the T-type cytoplasm is found in tetraploid *tuberosum* potatoes (Smyda-Dajmund et al.^2020^; Bradshaw 2021). We report for the first time DEGs in potato tubers with T- and D-type cytoplasm in response to cold treatment. Two potato diploid progenies derived from reciprocal crosses of parental clones that differed in cytoplasm type were used. We recognized DEPs associated with amyloplasts and mitochondria isolated from tubers characterized by light and dark chip colour after cold storage. Additionally, the tuber organelle levels were evaluated.

## Materials and methods

### Plant material and chip colour assessment

Material was collected from reciprocal crosses of the two diploid parental clones, DG12-3/54 and DG11-313. Clone DG12-3/54 had a T-type cytoplasm, whereas clone DG11-313 had a D-type cytoplasm (Supplementary Fig. S1). Both parental clones were interspecific *Solanum* hybrids containing *S. tuberosum*, *S. acaule S. chacoense*, *S. gourlayi*, *S. yungasense*, *S. verrucosum, S. demissum, S. stoloniferum, S. phureja* and *S. microdontum* in their pedigree. A family of 103 F1 individuals was obtained from the cross DG12-3/54 × DG11-313 (population T). For the cross DG11-313 × DG12-3/54 (population D), there were 120 F1 individuals. Plants were first sprouted for two weeks in the sprouting chamber, and then planted in tents as described in Sołtys-Kalina et al. (2015). Tubers of the parental and progeny plants were evaluated for chip colour after harvest (AH) and 3 months of cold storage at 4 °C (CS). Tubers were fried in three replications per genotype. For each replication four slices of each of two potato tubers were fried. Visual assessment of colour on a scale from 1 (dark) to 9 (light) was used as described by Jakuczun et al. (1995). For proteomic and transcriptomic studies, and detection of the organelle level, F1 individuals of CS tubers were selected.

### Construction of bulk samples

A pooling approach can facilitate findings of molecular factors associated with the trait of interest (Zou et al. 2016). We prepared bulk samples with contrasting chip colour to minimize the background effect on the nucleic acid and protein expression profiles. Two types of bulk samples collected after CS were constructed, each with three biological replications. Each bulk included samples from 2 to 3 different F1 individuals. Bulks A4 (bulk A after storage at 4 °C) and E4 were characterized by light chip colour after CS; bulks B4 and F4 had F1 individuals with dark chip colour after CS. Bulks A4 and B4 were made of population T, whereas bulks E4 and F4 were made of population D. In addition, reducing sugars were extracted from 0.3 g frozen tubers for both CS bulk samples and the corresponding samples collected after harvest, using a d-fructose/d-glucose assay kit (K-FRUGL, Megazyme).

### Preparation of crude amyloplast fraction from tubers

Amyloplast fractions were extracted according to the protocol described by Stensballe et al. (2008) with some modifications. Three slices from the middle section of tubers, approximately 2 g each, were placed in a Petri dish on ice containing a 10 ml of the isolation buffer and slightly chopped with a razor blade. The homogenate was filtered twice using 1 cm cotton swabs inside funnel. The homogenate was overlaid on 20 ml isolation buffer, containing 2% w/v Histodenz™ (Merck Life Science, Darmstadt, Germany), which was placed on a 7 ml 1% (w/v) agar pad (Merck Life Science) in a 50 ml tube. Centrifugations were carried out as described by Stensballe et al. (2008).

### Isolation of tuber mitochondrial fraction

Mitochondrial fraction from potato tubers was prepared according to the protocol described by Koszela-Piotrowska et al. (2009), with some modifications. First, 600 g of washed and peeled potato tubers was homogenized in a Braun type juice extractor (Germany). After homogenization, an equal volume of ice-cold extraction medium (0.35 M mannitol, 10 mM NaH_2_PO_4_/Na_2_HPO_4_ buffer pH 7.2, 2 mM EDTA, 2.9 mM cysteine, 0.1% BSA) was added to the homogenate. The extract was kept for 5 min on ice for starch sedimentation and then filtered through two layers of cheesecloth. The homogenate was centrifuged at 1500*g* for 10 min at 4 °C. The supernatant was transferred to new tubes and centrifuged at 11,000*g* for 15 min at 4 °C. The obtained pellet containing the crude mitochondrial fraction was washed with 10 ml medium consisting of 0.35 M mannitol, 10 mM NaH_2_PO_4_/Na_2_HPO_4_ buffer pH 6.8 and 0.1% BSA and transferred to 25 ml of a 21% Percoll continuous gradient. Mitochondrial fraction was centrifuged at 28,616*g* for 45 min. The purified mitochondrial layer was collected and washed twice in wash buffer (0.35 M mannitol, 10 mM MOPS buffer pH 6.8, 1 mM EDTA). The last centrifugation was conducted at 21,000*g* for 5 min at 4 °C. Mitochondrial pellet was re-suspended in 1 ml of ice-cold wash buffer and frozen in liquid nitrogen.

### RNA-seq analysis and Gene Ontology enrichment of DEGs

RNA was isolated from progeny tubers of populations T and D after CS. Isolation was performed according to Chomczynski and Sacchi (1987) protocol using TRIzol reagent. Briefly, to 0.1 g of ground tubers, 1 ml of TRIzol reagent was added. The extract was processed twice in chloroform. The RNA was precipitated in 0.3 ml of salt solution (0.8 M sodium citrate and 1.2 M sodium chloride) and 0.3 ml of isopropanol and suspended in sterile water. To degrade double-stranded and single-stranded DNA contaminants, RNA was treated with DNase I (Thermo Scientific, Waltham, MA, USA). The RNA quantity and integrity were examined using a Bioanalyzer 2100 (Agilent Technologies, Santa Clara, CA, USA). The Dynabeads^®^ mRNA Purification Kit for mRNA enrichment (Ambion, Waltham, MA, USA) was used for mRNA isolation and cDNA libraries were prepared using the MGIEasy RNA Directional Library Prep Set (MGI), both according to the manufacturer’s protocols.

The sequencing of cDNA libraries was performed by Genomed^®^ (Warsaw, Poland) on the BGISEQ-500 sequencing platform (BGI Genomics, Shenzhen, China). The obtained 100-bp paired-end reads were then subjected to qualitative filtering (adaptor sequences and low-quality reads). The quality control of sequenced reads is presented in Supplementary Table S1. Then, the index of the reference genome (https://www.ncbi.nlm.nih.gov/assembly/GCF_000226075.1) was built using Bowtie v2.1.0, and clean reads were aligned to the reference genome using TopHat v2.0.9 (Broad Institute, Boston, MA, USA) with the option ‘unstranded’. Next, to count the read number mapped to each gene, HTSeq v0.5.3 was used with the option of no differentiation for the strand of the transcript (-stranded = no). DEGs were identified by the DESeq package. The full name of gene products was annotated according to BlastX nomenclature. The TopGO package was used to enrich the gene set of GO terms (BlastX). To extract the significant GO categories, Fisher’s exact test was performed.

### Protein preparation and mass spectrometry

Amyloplast and mitochondrial protein extracts were resuspended in 25 mM ammonium bicarbonate with 0.4% SDS and sonicated for 30 min. The total protein content was measured using the bicinchoninic acid assay (Smith et al. 1985). For each bulk, 300 µg of the protein samples was prepared by Filter Assisted Sample Preparation (FASP), and analysed using nano-liquid chromatography coupled with tandem mass spectrometry (LC–MS-MS/MS) in the Laboratory of Mass Spectrometry, IBB PAS, Warsaw, to detect DEPs as described by Lebecka et al. (2019).

### DNA extraction and qPCR for determination of organelle DNA content

Total DNA was extracted from 0.15 g of potato tubers using a Food-Extract DNA Purification Kit (EURx, Gdansk, Poland). DNA quality and quantity were assessed as described in Niu et al. (2019). The relative quantification of organelle DNA, plastid DNA (ptDNA) and mitochondrial DNA (mtDNA), in comparison with nuclear DNA (nucDNA) was performed using RT HS-PCR Mix EvaGreen^®^ (A&A Biotechnology, Gdansk, Poland) and a Lightcycler 480 II System (Roche, Basel, Switzerland). The PCR conditions were 95 °C for 3 min followed by 40 cycles of 95 °C for 10 s, 60 °C for 30 s, and 72 °C for 30 s, and then a melting curve was prepared. The PCR primer pairs for ptDNA and nucDNA were used as described by Niu et al. (2019). Mitochondrial DNA marker from the cytochrome oxidase subunit III gene (accession number AF280607) was amplified using forward primer (coxIIIf) 5′-GTTTTACTAGGCGCGATAGA-3′, and reverse primer (coxIIIr) 5′-GTAGGATGGTTCACTGGAGA-3′. Organelle DNA content was evaluated in four types of progeny samples, recorded as: A′4, B′4, E′4 and F′4. For each sample, 15 biological replications were scored.

## Results

### Chip colour and reducing sugar content for AH and CS

The parental clones DG12-3/54 (T-type) and DG11-313 (D-type) were characterized by light chip colour for both AH and CS. The mean values for chip colour in the parents were 8.5 and 8.3 for AH, and 8.2 and 7.7 for CS, respectively. Progeny plants were used for construction of the bulks: A4 and B4 for population T and E4 and F4 for population D. All bulks had light chip colour and low reducing sugar content for AH (scores 7.9–8.5 and 0.0–22.0 mg/100 g FW, respectively). For CS, bulks A4 and E4 had light chip colour and low reducing sugar content (scores 7.7–7.8 and 1.0–10.2 mg/100 g FW, respectively), whereas B4 and F4 had dark chip colour (score 5.6–6.0) and reducing sugar content in the range of 30.8–245.0 mg/100 g FW (Supplementary Table S2).

### Sequencing data, detection of DEGs

**Fig. 1:**
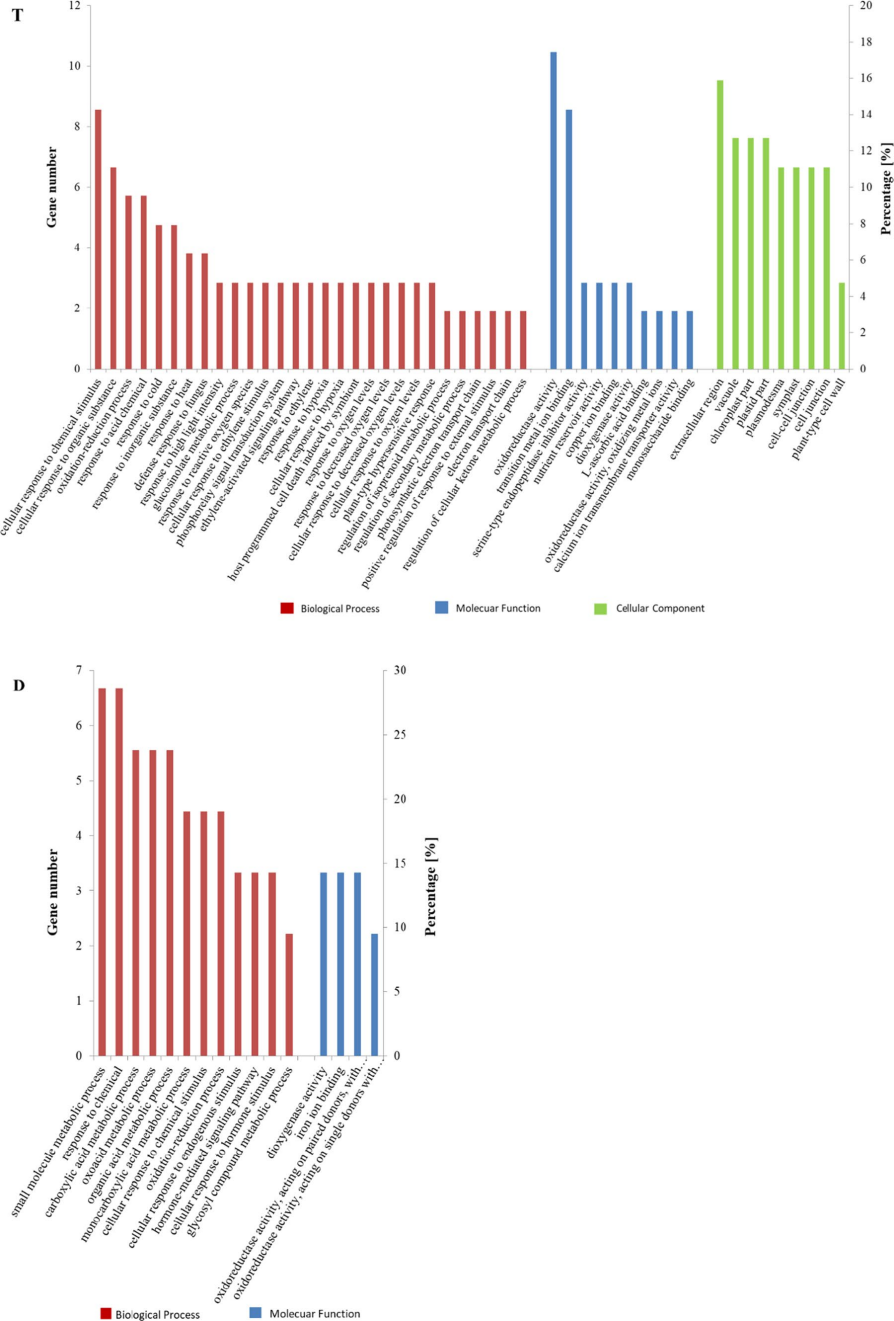
Gene ontology annotation of DEGs between RNA pools from tubers of light and dark chip colour in the T and D populations. Bar graphs represent annotation of DEGs in three categories: biological process (BP), molecular function (MF) and cellular component (CC). The *X*-axis represents GO terms: left *Y*-axis represents the number of DEGs annotated in each GO term; right *Y*-axis shows the percentage of DEGs, which were annotated in each GO term. None of the GO terms was statistically significant in CC (Fisher elim. ≤ 0.05) in population D

Altogether, over 745 million reads were generated, with the number of RNA-seq reads per library ranging from 30.2 to 31.7 million after filtering impurities (Supplementary Table S1). Finally, 23 250 and 24 192 genes were identified by alignment of the filtered reads and a potato reference genome (https://www.ncbi.nlm.nih.gov/assembly/GCF_000226075.1) in populations T and D, respectively. All raw and processed data have been deposited in the GEO database (NCBI) under the link: https://www.ncbi.nlm.nih.gov/geo/query/acc.cgi?acc=GSE184141. When B4 (dark chip colour) was compared to A4 (light chip colour), 48 DEGs were identified with *P* value < 0.05 and Log2 fold change ≥ 2.0 or ≤ − 2.0: 18 genes were upregulated and 30 were downregulated. For the comparison F4 vs. E4, the corresponding values were 6 and 9 (Table 1). The DEGs *ethylene-responsive transcription factor* (LOC102601547 and LOC102605338), *putative late blight resistance protein homolog R1B-23* (LOC102589432), and ATP*-dependent zinc metalloprotease FTSH* (LOC102603811) in population T possess the functionally corresponding analogs recognized in population D: *ethylene-responsive transcription factor* (LOC102591018), *putative late blight resistance protein homolog R1B-23* (LOC102581752), and *ATP-dependent zinc metalloprotease FTSH* (LOC102599039). All significantly DEGs are listed in Supplementary Table S3. The significant DEGs were aligned to the Gene Ontology (GO) database (Supplementary Table S4). For B4 vs. A4 data, the GO terms were assigned to the categories of biological process (BP, 29), molecular function (MF, 10) and cellular component (CC, 9). The GO annotation assigned the following GO terms associated with plastids: CC chloroplast part (GO:0044434) and plastid part (GO:0044435), each accounting for 12.7% of the GO terms (Fig. 1). Functional analysis of F4 and E4 placed the 21 DEGs into 16 GO terms, 12 in BP and 4 in MF (Fig. 1). None of the GO terms was statistically significant in CC (Fisher elim. ≤ 0.05). Only 3 common GO terms were found in the comparison of B4 vs. A4 and F4 vs. E4: cellular response to chemical stimulus (GO:0070887), oxidation–reduction process (GO:0055114) and dioxygenase activity (GO:0051213).

**Table 1:** List of DEGs recognized in comparison of bulks B4 *vs*. A4 (population T) and F4 vs. E4 (population D), obtained by using a cut-off (FDR < 0.05; Log2 fold change ≥ 2.0 or Log2 fold change ≤ − 2.0)

Locus	Gene name^c^	Log2 FC^b^	FDR
Bulks^a^			
B4 vs. A4			
LOC107058116	Putative calcium transporting ATPase 13. plasma membrane type	10.75	0.002
LOC102587258	Calcium binding allergen Ole e 8	3.83	0.000
LOC102592421	Metalloendoproteinase 3 MMP	3.44	0.000
LOC102586420	Dehydration responsive element binding protein 3	3.41	0.000
LOC102577806	*Beta amylase 3. chloroplastic*	3.19	0.041
LOC107062125	*pEARLI1like lipid transfer protein 3*	3.17	0.017
LOC102594756	*pEARLI1like lipid transfer protein 1*	2.87	0.010
LOC102601547	Ethylene responsive transcription factor ERF025	2.69	0.003
LOC102605338	Ethylene responsive transcription factor ERF025	2.41	0.010
LOC102600563	Lascorbate oxidase homolog	2.39	0.020
LOC102581894	*Monocopper oxidase like protein SKU5*	2.39	0.024
LOC102598886	Lysine rich arabinogalactan protein 18	2.37	0.003
LOC102585755	Dehydration responsive element binding protein 1F	2.35	0.011
LOC102601260	BUD13 homolog	2.22	0.001
LOC102593728	Probable linoleate 9S lipoxygenase 5	2.15	0.011
LOC102585650	Receptor like kinase TMK3	2.14	0.002
LOC102600693	Putative calcium transporting ATPase 13. plasma membrane type	2.11	0.040
LOC102599523	Cathepsin E	2.04	0.004
LOC102601381	Probable inactive patatin 3 Kuras 1	− 2.04	0.035
LOC102605260	Patatin13	− 2.07	0.017
LOC102589432	Putative late blight resistance protein homolog R1B23	− 2.12	0.002
LOC102586248	Protein RSI1	− 2.13	0.001
LOC102583050	Uncharacterized oxidoreductase Rv0769	− 2.19	0.000
LOC102595780	Wound induced basic protein	− 2.21	0.006
LOC102598955	Phospholipase A1 II delta	− 2.26	0.015
LOC102603811	*ATP dependent zinc metalloprotease FTSH 6. chloroplastic*	− 2.43	0.010
LOC102602628	E3 ubiquitin protein ligase MPSR1	− 2.46	0.039
LOC102586828	Cell wall / vacuolar inhibitor of fructosidase 1	− 2.48	0.006
LOC102589723	18.2 kDa class I heat shock protein	− 2.53	0.020
LOC102596508	*Temperature induced lipocalin 1*	− 2.55	0.015
LOC102604381	*ATPase expression protein 2. mitochondrial*	− 2.57	0.005
LOC102596945	Octanoyl transferase	− 2.71	0.010
LOC102601889	G2 specific protein kinase nim1	− 2.86	0.035
LOC102602095	Carboxymethylenebutenolidase homolog	− 2.90	0.010
LOC102578969	*26.5 kDa heat shock protein. mitochondrial*	− 3.15	0.003
LOC102577542	Aminocyclopropane 1 carboxylate oxidase 1	− 3.26	0.000
LOC102578786	*17.6 kDa class I heat shock protein*	− 3.28	0.017
LOC102589396	17.4 kDa class I heat shock protein	− 3.31	0.003
LOC102601494	22.7 kDa class IV heat shock protein	− 3.40	0.031
LOC102590753	Putative late blight resistance protein homolog R1A6	− 3.55	0.030
LOC102603518	22.7 kDa class IV heat shock protein	− 3.59	0.001
LOC102581269	Proteinase inhibitor type 2 CEVI57	− 3.65	0.005
LOC102577463	Naringenin 2 oxoglutarate 3 dioxygenase	− 3.70	0.003
LOC102599091	Very long chain aldehyde decarbonylase GL15	− 4.24	0.001
LOC102591853	*Small heat shock protein. chloroplastic*	− 4.88	0.000
LOC107059978	Integrase	− 5.09	0.041
LOC102602963	*Protein PROTON GRADIENT REGULATION 5. chloroplastic*	− 7.06	0.010
LOC102596598	Acylphosphatase	− 7.53	0.004
F4 vs. E4			
LOC102585245	Two component response regulator ARR15	7.07	0.015
LOC102599039	ATP dependent zinc metalloprotease FtsH	4.19	0.004
LOC107061761	Telomere repeat binding factor 1	4.09	0.033
LOC102591018	Ethylene responsive transcription factor ERF012	3.29	0.009
LOC102601716	Zerumbone synthase	3.20	0.020
LOC102596122	*Linoleate 13 Slipoxygenase 21. chloroplastic*	2.50	0.018
LOC102604838	V type ATP synthase subunit I	− 2.00	0.020
LOC102588395	*Proline dehydrogenase 1. mitochondrial*	− 2.06	0.023
LOC102585163	Cytochrome P450 94B3	− 2.26	0.004
LOC107062354	Uridine 5'monophosphate synthase	− 2.60	0.004
LOC102589278	Miraculin	− 2.74	0.008
LOC102581752	Putative late blight resistance protein homolog R1B23	− 2.87	0.024
LOC102602046	4.5DOPA dioxygenase extradiol	− 3.17	0.018
LOC102602308	Hyoscyamine 6 dioxygenase	− 5.60	0.004
LOC102601303	Cellulose synthase like protein G1	− 6.37	0.004

^a^Bulks A4 and E4 represent light chip colour genotypes; B4 and F4 represent dark chip colour genotypes

^b^Log2FC estimated fold change

^c^According to Blastx or Blastp name; italics—genes with direct GO annotation to plastids or mitochondria (based on CC category)

### Detection of DEPs

We employed an LC–MS-MS/MS system to detect DEPs in the amyloplast and mitochondrial fractions. For the amyloplast fractions, 2597 proteins were annotated. For the mitochondrial fractions, the corresponding value was 3485. The UniProt database (http://www.uniprot.org/) was used for the annotation of amyloplast-associated and mitochondria-associated proteins. In the T-type cytoplasm, only 2 amyloplast-associated and 5 mitochondria-associated DEPs with more than 2-fold change (*q* < 0.05) in expression level were detected (Table 2). Catechol oxidase (M1BMR6) had the highest level in bulk B4, whereas alpha-1,4 glucan phosphorylase L-1 isozyme (P04045) recorded higher abundance in bulk A4. Of the 5 DEPs isolated from the T-type cytoplasm and predicted as mitochondrial, 4 proteins: sorting and assembly machinery (Sam50) protein (M1CZK6), malate dehydrogenase (M1BPZ5), ascorbate peroxidase (M1A6L9) and endoplasmin homolog (M1ALZ6) were upregulated in bulk B4, whereas the mitochondrial small heat shock protein (M1A0Z7) was upregulated in bulk A4 (Table 2). Of 37 DEPs in the D-type cytoplasm, predicted to be mitochondria-associated, there were 36 downregulated DEPs in bulk F4 (Table 2). The lists of DEPs with unknown localization or possessing additional cellular compartment annotation according to the UniProt data are presented in Supplementary Tables S5 and S6.

**Table 2:** List of amyloplast-associated and mitochondria-associated DEPs identified in cold-treated potato tubers possessing T- and D-type of cytoplasm, based on the UniProt database (http://www.uniprot.org/)

Accession number^a^	Protein name	Fold change^b^	*q* value	#^c^
T-type cytoplasm		B4/A4		
Amyloplast-associated proteins				
M1BMR6	Catechol oxidase	2.1	0.00579	17
P04045^e^, ^f^	Alpha-1,4 glucan phosphorylase L-1 isozyme	0.49	0.00021	95
Mitochondria-associated proteins				
M1CZK6^e^	Sorting and assembly machinery (Sam50) protein	6.37	0.00006	37
M1ALZ6^e^	Endoplasmin homolog	3.77	0.00018	45
M1A6L9^e^	Ascorbate peroxidase	3.49	0.03104	12
M1BPZ5^e^	Malate dehydrogenase	2.39	0.03169	11
M1A0Z7	Mitochondrial small heat shock protein	0.35	0.00006	46
D-type cytoplasm		F4/E4		
Mitochondria-associated proteins				
M1ALZ6^e^	Endoplasmin homolog	7.76	0.00004	45
M1B744	Succinate dehydrogenase [ubiquinone] flavoprotein subunit^d^	0.47	0.00004	137
M1AEF2^e^	l-Galactono-1,4-lactone dehydrogenase protein	0.43	0.01498	47
M1AGZ2	Pentatricopeptide repeat-containing protein	0.42	0.02927	45
M1C0N9	Sulfurtransferase	0.37	0.03869	26
M0ZWY7, M1B641	Elongation factor Tu	0.33	0.00004	79
M1CHE3	Isocitrate dehydrogenase [NAD] subunit^d^	0.32	0.00094	43
M1BT60	CBS domain-containing protein	0.31	0.00004	38
M1AUM5^e^, M1AUM6	Gamma aminobutyrate transaminase isoform1	0.28	0.00827	46
P52903^f^	Pyruvate dehydrogenase E1 component subunit alpha^d^	0.26	0.00025	49
M1B8S4^e^, ^f^	Malate dehydrogenase^d^	0.26	0.01475	32
M1ATY8^e^	Chaperonin CPN60-2	0.25	0.00004	51
M1AQ24	Methylmalonate-semialdehyde dehydrogenase (CoA acylating)	0.25	0.00197	62
M1BM43	Acetyltransferase component of pyruvate dehydrogenase complex^d^	0.24	0.00004	55
M1BCU6	Succinate–CoA ligase [ADP-forming] subunit alpha^d^	0.24	0.02991	44
M0ZG23, P37225	Malic enzyme^d^	0.22	0.00004	107
M1C4X8	Alcohol dehydrogenase	0.22	0.0037	35
M1BYP7	Uncharacterized protein	0.22	0.02836	18
P37221	NAD-dependent malic enzyme 62 kDa isoform^d^	0.21	0.00004	88
M1A0Z7^f^	Mitochondrial small heat shock protein	0.21	0.00095	43
M1BEG6	NAD-dependent isocitrate dehydrogenase^d^	0.21	0.0081	33
M1CL95^e, f^	Chaperonin-60 kD, ch60	0.19	0.00004	70
M1BCZ5, P54260	Aminomethyltransferase	0.19	0.00008	52
M1A028	Dihydrolipoyl dehydrogenase	0.18	0.00004	50
M1C9T0	Aldehyde dehydrogenase	0.17	0.00004	75
M1AYY7^e^	Aminotransferase	0.13	0.00004	100
M1A6G8^e^	Heat shock protein	0.13	0.00004	62
M1CL86	Dihydrolipoyl dehydrogenase	0.13	0.00008	45
Q43175, M1CTV7	Citrate synthase, mitochondrial^d^	0.12	0.00004	97
M1AX44^e^, ^f^	Malate dehydrogenase^d^	0.12	0.00004	74
M1BDU1	Superoxide dismutase	0.11	0.00004	77
M1BSG4^f^	Succinate–CoA ligase [ADP-forming] subunit beta^d^	0.11	0.00004	63
M1AFA9	Glutamate dehydrogenase	0.1	0.01178	30
M0ZI98^e^, Q07511^e^	Formate dehydrogenase^d^	0.09	0.00004	167
M1AQ30, M1AQ31^e^, Q08276^e^	Heat shock 70 kDa protein	0.08	0.00178	21
M1D6K2^f^	1-Pyrroline-5-carboxylate dehydrogenase 1	0.06	0.00004	125
M1CTR3^e^	Aconitate hydratase^d^	0.06	0.00004	112

^a^Accession number according to the Universal Protein Resource database (UniProt) (uniprot.org)

^b^Fold change is expressed as the relative abundance of proteins in comparisons of B4/A4 and F4/E4 (*q* ≤ 0.05)

^c^Number of peptides matched to predicted protein sequence

^d^Enzyme associated with the tricarboxylic acid cycle

^e^Also distributed in other cellular compartments using UniProt

^f^Posttranslational modifications of peptides (phosphorylation and/or ubiquitination and/or not oxidation)

### qPCR for organelle DNA content

**Fig. 2:**
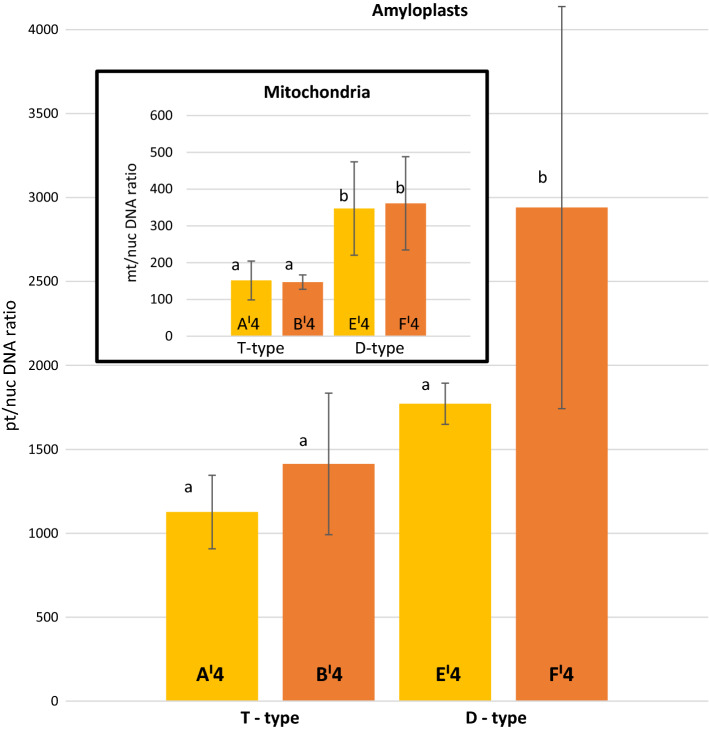
The mt/nucDNA ratios and pt/nucDNA ratios for samples from potato tubers characterized by the T- and D-type cytoplasm that had light (samples A′4 and E′4) and dark (B′4 and F′4) chip colour after cold storage. The bar represents SD of 15 samples

The DNA molecules for the plastid, mitochondria and nucleus were evaluated in the tuber progeny plants of populations T and D after CS. The F′4 and B′4 samples had pt/nucDNA ratios 2.08-fold (*P* < 0.05) and 1.57-fold (*P* < 0.05) higher than those of samples E′4 and A′4, respectively. The mt/nucDNA ratio at E'4 was 2.38-fold (*P* < 0.05) higher than that at A′4. For samples F'4 and B′4 the corresponding value was 2.36-fold (*P* < 0.05) (Fig. 2).

## Discussion

There are many examples for cytoplasmic effects on disease resistance and agronomic traits in potato. D-type cytoplasm is associated with late blight resistance and male sterility (Sanetomo and Gebhardt 2015). The T-type seems to be related with higher potato tuber yields, which is strongly associated with tuber starch content (Maris 1989; Hosaka and Sanetomo 2012). In our study, much higher glucose and fructose contents were observed for bulks B4 and F4 characterized by dark chip colour after CS than in the bulks that had light chip colour (Supplementary Table S2). This may reflect an association of reducing sugar level with potato chip colour. The strong correlation between chip colour and reducing sugar content has been documented (Scheffler et al. 1992; Werij et al. 2012). More than 90% of the chip color variation could be related to the reducing sugar content in potato tubers (Hughes and Fuller 1984).a Our findings suggest that T- and D-types of cytoplasm might influence sugar accumulation in cold-stored potato tubers in different ways. We showed that within the T- and D-type cytoplasm, there are different sets of DEGs in potato tubers characterized by light and dark chip colour. Based on *P* value < 0.05 and Log2 fold change ≥ 2.0 or ≤ − 2.0, of 63 DEGs selected, there were DEGs encoding 3 proteins whose functions remained related in T and D populations. In cold-stored potato tubers, a relationship between the respiration rate, sugar content and ATP levels has been reported (Blenkinsop et al. 2004; Pinhero and Paliyath 2005). In population T, the *putative calcium-transporting ATPase 13, plasma membrane-type* (LOC107058116) gene recorded the highest level of expression (upregulated 10.75 Log2 fold change). This is a magnesium-dependent enzyme that catalyses the hydrolysis of ATP coupled with the translocation of calcium from the cytosol out of the cell or into organelles. Gounaris (2001) postulated a central role of the intracellular calcium ion concentration generated by the inhibition of ATPase activity in sugar accumulation in low temperature stressed plant tissues.

The relationship between mRNA and protein levels is complex. The proteome reflects a dynamic balance among posttranscriptional, translational and protein modification/destruction processes (Vogel and Marcotte 2012; Zapalska-Sozoniuk et al. 2019). Thus, mRNA is an imperfect indicator of protein abundance and activity. Only 2 amyloplast-associated DEPs indicating more than 2-fold changes (*q* < 0.05) in expression level were identified: catechol oxidase and alpha-1,4 glucan phosphorylase L-1 isozyme. They were expressed in the T-type cytoplasm. Catechol oxidases belong to the family of polyphenol oxidases (PPOs) (Molitor et al. 2016). A strong inhibitory effect of reducing sugars on potato PPOs has been detected (Lee and Park 2005). Glucan phosphorylases are important enzymes in carbohydrate metabolism in plants (Rommens et al. 2006). In our study, lower expression level of alpha-1,4 glucan phosphorylase L-1 isozyme was revealed in bulk B4 (tubers with dark chip colour) compared to bulk A4 (light chip colour samples). Sucrose synthase (SuSy) is a glycosyl transferase enzyme that plays an important role in sugar metabolism in potato tubers (Sowokinos 2001; Blenkinsop et al. 2004; Zhang et al. 2017). SuSy proteins are mainly located in the cytosol but have also been localized to plant organelles (Stein and Granot 2019). We speculate that two protein species of SuSy (M0ZT40, P10691) identified in our study in the T-type cytoplasm (Supplementary Table S5). might be associated with potato amyloplasts.

A significantly higher number of DEPs predicted as mitochondrial were identified in the mitochondria-enriched fractions isolated from tubers with D-type cytoplasm than with T-type cytoplasm (37 vs. 5). Two common proteins were found: the endoplasmin homolog (M1ALZ6) and mitochondrial small heat shock protein (M1A0Z7). The first one increased significantly, while the second decreased in bulks B4 and F4 compared to bulks A4 and E4 (Table 2). At present, the significance of this finding is unclear, but it is interesting that both proteins have chaperone activity. M1ALZ6 is a member of the heat shock protein 90 (Hsp90) family, which is essential for survival of eukaryotes under certain physiological and stress conditions (di Donato and Geisler 2019). We showed previously that the *Hsp90* gene is a candidate gene capable of influencing the chip colour of potato tubers (Sołtys-Kalina et al. 2015). In the T-type cytoplasm, the sorting and assembly machinery (Sam50) protein (M1CZK6) showed a large increase in expression level (6.37-fold) in bulk B4 (Table 2). Sam50 is a component of the mitochondrial outer membrane protein import machinery (Duncan et al. 2011). Among 37 DEPs recorded for mitochondria in the D-type cytoplasm, as many as 14 enzymes are involved in tricarboxylic acid cycle metabolism (Table 2). This suggests that the respiratory pathway may play an essential role in the response of D-type potato tubers to cold stress. Two isoforms of malate dehydrogenase (M1B8S4 and M1AX44) revealed posttranslational modifications and showed downregulated expression patterns in bulk F4. In contrast, a different isoform of malate dehydrogenase (M1BPZ5) was upregulated in bulk B4 of the T-type cytoplasm. (Table 2). It will be interesting to investigate the specific function of malate dehydrogenase isoforms in sugar accumulation in potato tubers possessing T- and D-type cytoplasm.

We showed that the pt/nucDNA ratio was higher in D-possessing tubers after cold storage than in T-type progeny. Autophagy is an important process for degrading proteins and organelles in all eukaryotic cells. Plant autophagy is critical for maintaining cellular homeostasis under normal conditions and is upregulated during a wide range of abiotic/biotic stresses (Su et al. 2020). It has been postulated that there is an interplay between sugar signaling and autophagy pathways in plants (Janse van Rensburg et al. 2019). Therefore, a protective effect of reducing sugars in potato amyloplasts in the D-type cytoplasm during low temperature treatment should be considered.

### *Author contribution statement*

KS and DSK performed most of the experiments and cowrote the manuscript; PSD performed analysis of the cytoplasm types; IWF contributed to chip colour evaluation; MHC contributed to isolation of the mitochondria fractions; HJ contributed to data analysis; WM conceived the idea, coordinated the project and wrote the manuscript.

## Supplementary Information

Below is the link to the electronic supplementary material.

Supplementary file1 (DOCX 917 KB)

Supplementary file2 (XLSX 13 KB)

Supplementary file3 (DOCX 96 KB)

Supplementary file4 (XLSX 17 KB)

Supplementary file5 (XLSX 31 KB)

Supplementary file6 (DOCX 16 KB)

Supplementary file7 (XLSX 22 KB)

## Abbreviations

AH: After harvest
CS: Cold storage at 4 °C
DEGs: Differentially expressed genes
DEPs: Differentially expressed proteins
mtDNA: Mitochondrial DNA
nucDNA: Nuclear DNA
ptDNA: Plastid DNA
