# Supplementary material for: Transcriptomic and proteomic data provide new insights into cold-treated potato tubers with T- and D-type cytoplasm
Source: Planta. 2022 Apr 5;255(5):97. doi: 10.1007/s00425-022-03879-2 (PMC8983635; doi:10.1007/s00425-022-03879-2)
Supplement: Supplementary file 1 — Supplementary file1 (DOCX 917 KB) [file 425_2022_3879_MOESM1_ESM.docx]

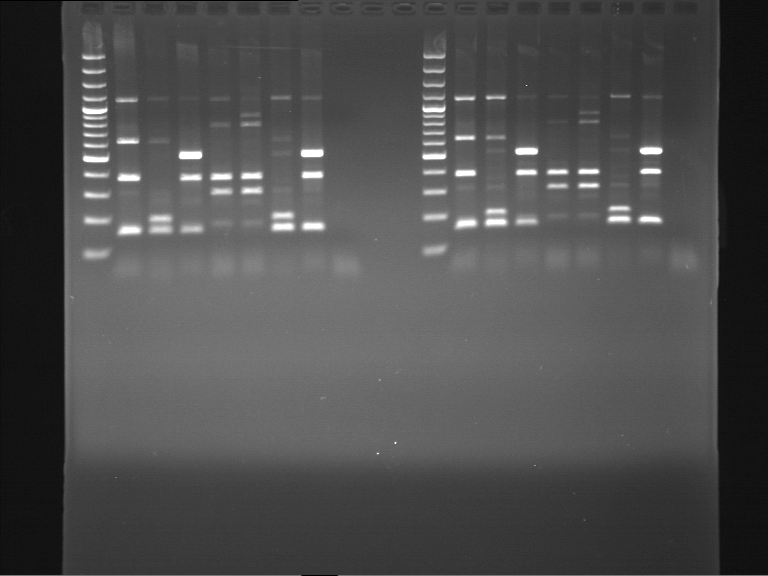


DG 12-3/54

DG 11-313

W

T

D

P

A

500 bp

200 bp

100 bp

Supplementary Fig. S1. Types of cytoplasm for parental clones DG 12-3/54 and DG 11-313 amplified in multiplex PCR and digested by *Bam*HI. Standards: wild species *S. × michoacanum* (W), cv. Early Rose (T), breeding line PW 363 (D), cultivated species *S. phureja* (P) and cv. Maris Piper (A). A 100-bp DNA ladder was used as a size marker.
