# Supplementary material for: Transcriptomic and proteomic data provide new insights into cold-treated potato tubers with T- and D-type cytoplasm
Source: Planta. 2022 Apr 5;255(5):97. doi: 10.1007/s00425-022-03879-2 (PMC8983635; doi:10.1007/s00425-022-03879-2)

Supplementary Table S2. Chip colour and reducing sugar content of bulks A4 and B4 (population T); E4 and F4 (population D). The scores represent average of the individual scores in the pooled samples (±SD) and range of variation for reducing sugar content [mg/100g FW]. AH – chip colour after harvest; CS – chip colour after three months of cold storage at 4°C.

|  | **AH** | **Range of variation glucose+fructose** | **CS** | **Range of variation glucose+fructose** |
| --- | --- | --- | --- | --- |
| **Bulks** |  |  |  |  |
|  |  |  |  |  |
| A4 | 8.5±0.7 | 0.0-13.5 | 7.8±0.5 | 1.0-5.0 |
| B4 | 8.0±0.6 | 4.0-22.0 | 5.6±0.6 | 30.8-131.4 |
| E4 | 8.4±0.4 | 7.1-12.0 | 7.7±0.2 | 2.5-10.2 |
| F4 | 7.9±0.8 | 0.0-8.7 | 6.0±0.4 | 71.0-245.0 |

Photographs of the different chipping colours, ranging from 4 (*dark*) to 9 (*light*).


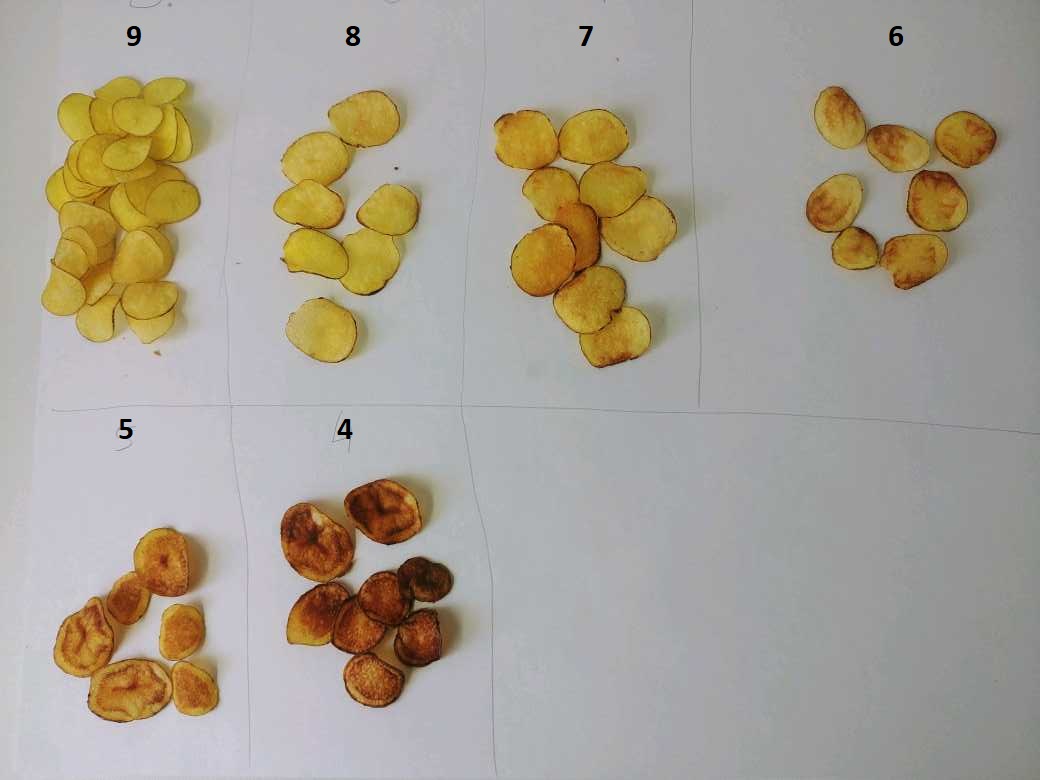

Supplement: Supplementary file 3 — Supplementary file3 (DOCX 96 KB) [file 425_2022_3879_MOESM3_ESM.docx]
