# Supplementary material for: Transcriptomic and proteomic data provide new insights into cold-treated potato tubers with T- and D-type cytoplasm
Source: Planta. 2022 Apr 5;255(5):97. doi: 10.1007/s00425-022-03879-2 (PMC8983635; doi:10.1007/s00425-022-03879-2)
Supplement: Supplementary file 6 — Supplementary file6 (DOCX 16 KB) [file 425_2022_3879_MOESM6_ESM.docx]

Supplementary Table S5. List of DEPs in additional cellular compartments identified in the amyloplast fractions isolated from cold-stored tubers possessing the T – and D – type of cytoplasm using a cut-off of fold change ≥2 and ≤ 0.5.

| **Accession number^a^** | **Protein description** | **Fold change^b^** | **q-value** | **#^c^** |
| --- | --- | --- | --- | --- |
| **T-type cytoplasm** |  | **B4/A4** |  |  |
| M1BD67, M1BD68 | Stem 28 kDa glycoprotein | 3.12 | 0.00021 | 29 |
| M1CPL5 | Short chain alcohol dehydrogenase | 2.94 | 0.02028 | 9 |
| M0ZT40, P10691 | Sucrose synthase | 2.4 | 0.00571 | 19 |
| M1CLR1 | Acyl carrier protein | 0.32 | 0.04671 | 5 |
| P30941 | Serine protease inhibitor 7 | 0.18 | 0.0227 | 9 |
| **D-type cytoplasm** |  | **F4/E4** |  |  |
| M1BUH4 | Elicitor-inducible protein EIG-J7 | 2.17 | 0.02722 | 9 |
| M1AKE5, P58519, P58520, P17979, P58521, P16348, Q03197* | Aspartic protease inhibitors | 0.47 | 0.00038 | 18 |
| Q9AVQ1, M1CCW0* | Divinyl ether synthase | 0.45 | 0.00038 | 27 |
| M1AHQ0, M1B9J7 | Histone H2A | 0.42 | 0.02408 | 8 |
| P30941 | Serine protease inhibitor 7 | 0.40 | 0.0017 | 10 |

^a^ Accession number according to UniProt

^b^ Fold change is expressed as relative abundance of proteins in ratios B4/A4 and F4/E4 (q ≤ 0.05)

^c^ Number of peptides matched to predicted protein sequence

*Ubiquitination of one or more peptides
